# Editorial: Can virtual reality be a solution for assessing and treating psychological symptoms caused by eating and weight disorders?

**DOI:** 10.3389/fpsyg.2023.1225454

**Published:** 2023-06-13

**Authors:** Julia Vázquez-de Sebastián, Dimitra Anastasiadou, Desiderio Cano Porras, Doron Friedman, Carmina Castellano-Tejedor, Pilar Lusilla-Palacios

**Affiliations:** ^1^Psychiatry, Mental Health and Addictions Research Group, Vall d'Hebron Research Institute, Barcelona, Spain; ^2^RE-FiT Barcelona Research Group, Vall d'Hebron Research Institute and Parc Sanitari Pere Virgili, Barcelona, Spain; ^3^Health and Sport Psychology PhD Program, Psychology Department, Autonomous University of Barcelona, Bellaterra, Spain; ^4^Department of Clinical and Health Psychology, Autonomous University of Barcelona, Barcelona, Spain; ^5^Department of Cognitive Science and Artificial Intelligence, Tilburg School of Humanities and Digital Sciences, Tilburg University, Tilburg, Netherlands; ^6^School of Communications, Reichman University, Herzliya, Israel; ^7^GIES Research Group, Basic Psychology Department, Autonomous University of Barcelona, Barcelona, Spain; ^8^Psychiatry Department, Vall d'Hebron University Hospital, Barcelona, Spain; ^9^Department of Psychiatry and Legal Medicine, Universitat Autònoma de Barcelona, Barcelona, Spain; ^10^Biomedical Network Research Centre on Mental Health (CIBERSAM), Barcelona, Spain

**Keywords:** eating and weight disorders, obesity, virtual reality, psychological treatment, psychotherapy

Recent scientific literature suggests that incorporating Virtual Reality (VR) into psychological interventions for Eating and Weight Disorders (EWD) has promising potential for addressing the root causes of these conditions beyond traditional weight control or weight recovery-focused strategies. For instance, VR may provide multisensory (e.g., visual, auditory, olfactory, and haptic cues) and extrinsic feedback, such as objective information regarding performance, or embodiment experiences. Thus, applying theory-driven choices in VR can contribute to increasing patients' motivation and adherence to clinical treatments, and can support clinicians to personalize interventions. Moreover, VR is emerging as a promising tool for patients, as it can facilitate the promotion of healthy behaviors, reduce negative body image concerns, address the internalization of weight stigma, and offer them the opportunity to safely and effectively confront adverse stimuli in controlled environments (Riva et al., 2020; Al-Rasheed et al., 2022).

One key question addressed in this Research Topic is whether the latest VR developments can be successfully implemented to enhance the efficacy of evidence-based psychological techniques for people living with EWD.

Studies included in this Research Topic suggest that VR has the capacity to create immersive and experiential platforms for delivering interventions in a life-like and fully engaging manner, which could ultimately improve clinical effectiveness (Anastasiadou et al.; Behrens et al.; Döllinger et al.; Harris et al.; van der Waal et al.). Harris et al. explored the effect of high levels of immersion and interactivity. Particularly, the authors analyzed the effects of VR food stimuli on participants' cravings, by adding olfactory and interaction cues within the virtual environment. This study concluded that VR could be a useful tool for exposure treatments; in this case, food cravings increased through the use of VR.

Additionally, immersive VR has the potential to enhance the understanding of the factors involved in EWD for both clinicians/researchers and patients. One important advantage is the ability to gather objective data on patients' eating behavior and emotional responses, providing valuable insights into the psychological mechanisms underlying obesity. For instance, van der Waal et al. used visuotactile stimulation in VR to explore the complex interplay between emotions and body-size perceptions among healthy participants, providing insights into participants' negative emotional responses toward avatars with obesity.

VR technology also helps to incorporate immersive psychoeducational cues and participatory environments to empower people with EWD to better manage their condition. Furthermore, the integration of Cognitive-Behavioral Therapy (CBT) and Motivational Interviewing (MI) with VR exposure to challenging situations, which may be difficult to recreate in the real world, can enhance clinical interventions. For instance, VR allows participants to maintain self-conversations using *embodiment* and *body-swapping* techniques, as in the SOCRATES project (Anastasiadou et al.). Such techniques consist on alternatively embodying two different avatars; one being the patient's look-alike avatar, and the other being a virtual counselor. This study shows that incorporating VR-driven *body-swapping* with MI, can promote healthier lifestyles among patients with obesity (Anastasiadou et al.). Another advantage of using VR in the treatment of EWD is the ability to address body image disturbances by allowing individuals to embody visually altered virtual bodies. Döllinger et al. developed a VR prototype system that employs personalized avatars with photorealistic features, which can be modulated in real-time. Such a system may enable people with obesity to engage in virtual scenarios that simulate real-world challenges related to body image concerns (Döllinger et al.). Hence, exposure to virtual situations can provide a safe and effective way to address negative feelings about body image, a critical factor in overweight and obesity. This approach can lead to improved body image acceptance and body awareness, thus helping individuals with EWD to develop new, healthier habits and attitudes (Ferrer-García and Gutiérrez-Maldonado, 2012).

Moreover, VR-based interventions offer the possibility to enhance engagement in treatment by providing a unique and impactful therapeutic experience. However, it is necessary to consider various factors influencing the user experience, such as familiarity with VR, individual characteristics like age and gender, and also, specific technological design and technical features, such as the quality of graphics and audio in the VR environment. Besides, by tailoring VR interventions to meet the specific needs and preferences of the target audience, researchers and designers can improve their effectiveness and implementation. Understanding all these factors is essential for optimizing the design and effectiveness of VR interventions for treating EWD in clinical practice (Behrens et al.).

Recent progress in generative Artificial Intelligence, and specifically breakthroughs with large language models (Brown et al., 2020), are also likely to facilitate further research and application in the development of VR systems as therapeutic tools to address EWD. First, the generation of VR scenarios, including personalized scenarios, is likely to become easier and cheaper due to automated generation of technical code and content (Pollak et al., 2023). Additionally, offline analysis of psychological textual content, such as MI transcripts, has become feasible (Wu et al., 2023). Finally, it is becoming easier to integrate intelligent virtual humans in psychological VR applications (Nakash et al., 2022), and these virtual humans are becoming increasingly sophisticated and competent in their dialogue capabilities (Chiu et al., 2015).

Considering the accelerated technological advance and the growing body of literature, interdisciplinary collaboration among researchers, clinicians, and VR designers is essential to ensure a successful transfer to the clinical implementation of VR in the treatment of EWD. Additionally, to ensure a transition into evidence-based practice, VR products should undergo testing and validation before clinical application, and the personal data and information of patients using VR products must be secure and protected. This will strengthen the quality of the VR technology and facilitate its incorporation into solutions for assessing and treating psychological symptoms associated with EWD.

In sum, improving the quality of future studies is crucial for evaluating the effectiveness of these interventions in different clinical populations considering their specific needs, and ensuring the transferability of these results in clinical practice.

## Author contributions

JV-dS, DA, DC, DF, and PL-P drafted the manuscript. CC-T provided critical revisions. All authors approved the final version of the manuscript.
